# Retrieval of dislodged coronary stent from left renal artery by gooseneck snare^☆^

**DOI:** 10.1016/S1674-8301(10)60064-4

**Published:** 2010-11

**Authors:** Chunjian Li, Zhijian Yang, Kejiang Cao

**Affiliations:** Department of Cardiology, the First Affiliated Hospital of Nanjing Medical University, Nanjing, Jiangsu 210029, China

**Keywords:** percutaneous coronary intervention, complications, stent, gooseneck snare, renal artery

## Abstract

A rapamycin-eluting stent was dislodged during attempt of implantation at the proximal right coronary artery, which was found by fluoroscopy to have migrated into the anterior trunk of the left renal artery. We chose a 5 mm diameter Amplatz gooseneck snare and successfully retrieved the lost stent from the lodging vessel.

Stent loss during percutaneous coronary intervention (PCI) is an infrequentl coronary event[1]–[6], and yet it is associated with an increased risk of complications[1],[2]. Most dislodged coronary stents were lost and retrieved within the coronary artery[5],[7]–[8]. Successful retrieval of a lost coronary stent from a renal artery has not been reported. In this report, we report the revival of a dislodged rapamycin-eluting stent during attempt of implantation at the proximal right coronary artery (RCA) after removal off the guide-wire. The lost stent was successfully retrieved by a gooseneck snare from the anterior trunk of the left renal artery.

## CASE REPORT

An eighty-year-old man was referred to our hospital due to exertional chest pain, which had been present over the previous two w. The patient had received a PCI for unstable angina pectoris 10 y before when a 2.75×28 mm bare metal stent was planted in the proximal RCA. Other relevant history included hypertension of 40 y and type II diabetes of 20 y.

**Fig. 1:**
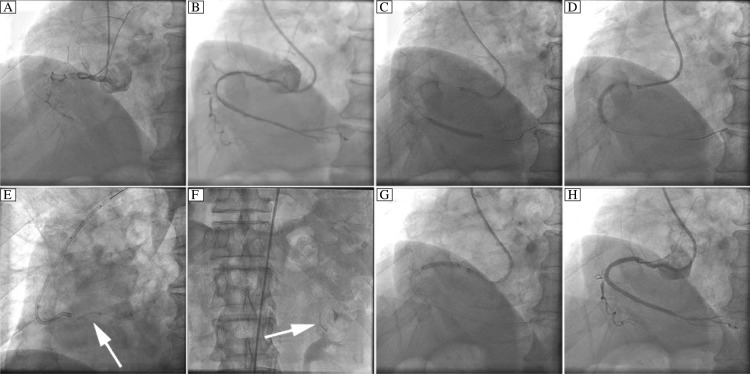
Percutaneous Coronary Intervention of the right coronary artery. A: right coronary artery (RCA) angiography. B: after pre-dilation. C: a 2.75×29 mm Partner stent was deployed at the distal RCA. D: a 2.75×24 mm Partner was deployed stent at the median RCA. E: dislodged stent. F: the dislodged stent was found in the anterior trunk of the left renal artery. G: a 3.0×21 mm Partner stent was deployed at the proximal RCA. H: final angiographic result. Arrow indicates stent.

**Fig. 2:**
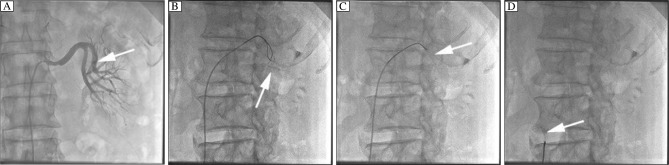
Retrieval of the dislodged stent. A: Dislodged stent was found in the anterior trunk of the left renal artery. Simultaneously, a 50% stenosis was found at the proximal left renal artery. B: A gooseneck snare was advanced to the lodging vessel. C: Dislodged stent was grasped by the snare. D: The stent was withdrawn from the renal artery. Arrow indicates stent.

Coronary artery angiography demonstrated that: (1) the proximal RCA was totally occluded (***Fig. 1A***); (2) the left anterior descending artery had 30%-50% stenosis in the proximal and the distal segments; (3) there was no lesion at the left main or left circumflex coronary artery.

PCI was performed to re-canalize the RCA through the right radial artery. A 6F XB RCA guide catheter was used to engage the ostium of the RCA, Balance Moderate Weight (BMW), Runthrough NS and Miracle 4.5 guide-wires were advanced in turn into the RCA. After extensive attempts, only the Miracle 4.5 wire was advanced across the occlusive lesion. However, as the 1.5 mm×15 mm Maverick balloon could not pass across the occlusion after several attempts, we decided to change the route of the procedure. Then, a 7F sheath was inserted into the right femoral artery and a 6F AL1 guide catheter was advanced to engage the ostium of the RCA, Miracle 4.5 wire was again successfully pushed across the occlusive lesion and positioned at the distal portion of the right atrioventricular branch. Fortunately, the 1.5 mm×15 mm Maverick balloon was managed to pass across the lesion this time. Subsequently, the target lesion was dilated with the 1.5 mm×15 mm Maverick and another 2.5 mm×20 mm Avita balloon, after which a diffused lesion was disclosed in the proximal, median and distal RCA (***Fig. 1B***).

Shortly afterwards, two rapamycin-eluting stents (2.75 mm×29 mm, 2.75 mm×24 mm Partner) were successfully deployed at the distal and median RCA (***Fig. 1C*** and ***1D***). But when we tried to plant a 3.0 mm×24 mm Partner stent at the proximal RCA, we encountered very strong resistance. More frustratingly, the guide catheter became disengaged from the RCA ostium and sprang uncontrollably into the aortic root when a strong push was applied to the delivering system, which subsequently pulled the guide-wire and the stent delivery system out of the RCA. We then attempted to pull back the stent-balloon assembly together with the guidewire, but the stent was blocked at the tip of the guide catheter and was suddenly dislodged from the balloon and slipped off the guide-wire. At the sight of what was happening, cineradiography was performed immediately and the image of the lost stent was captured while it was pulsating near the aorta root several seconds before it was flushed away (***Fig. 1E***).

Due to the stable condition of the patient, we were able to screen for the lost stent and then resume the coronary intervention. Fluoroscopy was performed from head to feet of the patient. As a result, a suspected image was found in the anterior trunk of the left renal artery, which was similar to the shape of the stent as captured after it was undeployed (***Fig. 1F***). Finally, a relatively shorter stent (3.0 mm×21.0 mm Partner) was successfully deployed at the proximal RCA with good angiographic result (***Fig. 1G*** and ***Fig. 1H***).

**Fig. 3:**
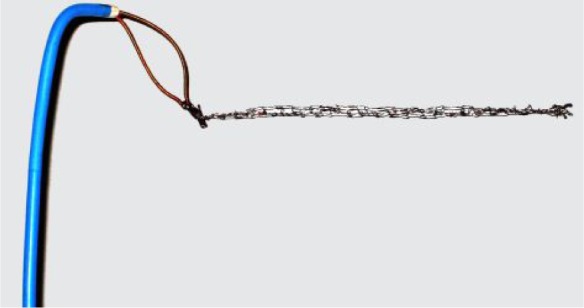
The gooseneck snare used for retniesal of dislodged stent

Thereafter, angiography of the left renal artery was performed, which showed that the suspected image was parallel to the edge of the lodging branch (***Fig. 2A***). Then, we chose a 5-mm Amplatz gooseneck snare, which was successfully positioned to the distal end of the suspected image under a 6F JR4.0 guide catheter. The image was proved to be of the lost stent as it moved while being pulled (***Fig. 2B***). The snare was then manipulated gently to pull the stent by the tip of the loop. After extensive attempts in the next 40 min, the stent suddenly bounded into the loop, and was successfully retrieved from the lodging vessel (***Fig. 2C, Fig. 2D***, and ***Fig. 3***). Angiography of the left renal artery was re-performed at the end of the procedure and no injury of the artery was found after retrieval of the stent. The patient did not experience any discomfort during the procedure, and was discharged three d later without any complication.

## DISCUSSION

The incidence of stent loss during PCI is reported to be only 0.32%[1], yet it is associated with an increased risk of complications[1],[2]. Although retrieval of lost stent from the coronary artery has been well described[5],[7],[8], only one source described a lost coronary stent which moved to the renal artery. The retrieval, however, was complicated by the stent being lost again and migrating to the left deep femoral artery[9].

**Fig. 4:**
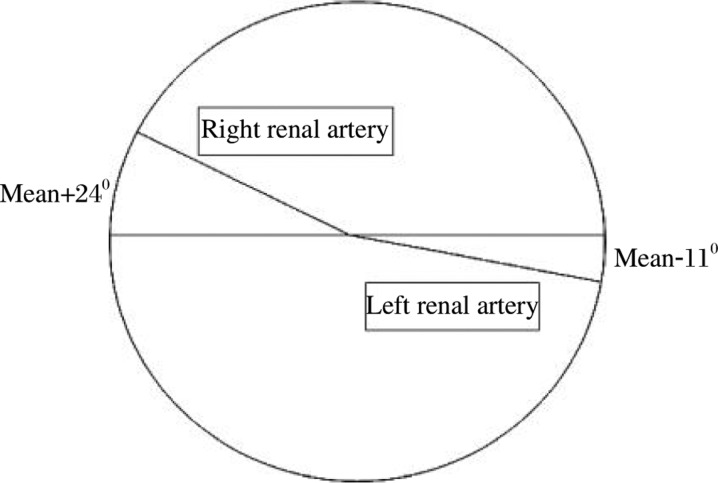
Angle of the origin of the right and left renal arteries from the aorta in the axial transverse section

Beregi *et al.*[10] found that the angle of the origin of the renal artery from the aorta in the axial transverse section averaged + 24^0^ on the right side and -11^0^ on the left side (***Fig. 4***). Similar results were reported by Turba UC *et al*.[11]. Accordingly, we speculate that the lost stent would have a tendency to migrate into the left renal artery owing to the different angles of the origin of the two renal arteries, and always move downward the direction of the weight applied to the stent.

It has been reported that stent losses occur with equal frequency in different coronary arteries and various locations within the coronary artery[1]. On the other hand, the presence of calcification and the severity of proximal angulation are two significant predictors of stent loss[1],[4]. In this case, both risk factors were present in the target lesion. However, severe stent restenosis in the patient was possibly another risk factor for the stent loss.

The gooseneck snares are made of nickel-titanium cables and produced with different loop sizes (5, 10, 15, 25, and 35 mm)[12]. When the wire is deployed, the loop is oriented at a right angle to the cable, and when pulled, the lost stent would be trapped between the wire and the outer catheter. Maintaining continuous traction prevents the lost stent from escaping and the entire system can be withdrawn. Obviously, the snare technique suits the lost stent that has moved out of the guide-wire as in this case.

We chose an Amplatz gooseneck snare 5 mm in diameter and successfully retrieved the lost stent from the left renal artery. several caveats were learned for this incidence: i) Once stent loss is noticed, immediate cineradiography is useful if the dislodged stent can be captured before disappearing from the screen. The recorded shape of the stent can serve as a reference to differentiate the image of the real lost stent from false images being screened while searching for the stent; ii) Performing control angiography of the lodging vessel before retrieval is helpful to test whether the suspected image is parallel to the vessel, by which the assessment of the suspect image of the lost stent would be further improved. Moreover, the angiography can serve as a baseline control for the post-retrieval image of the lodging artery if any complications were to occur; iii) The gooseneck snare is a useful apparatus for retrieving the lost stent. However, gentle operation and great patience are required when manipulating the snare system; iv) Re-performing angiography of the lodging vessel is necessary to make sure no injury such as dissection and rupture occurs after the retrieval.

Alternative techniques that can be used to retrieve the lost stent include balloon technique, two-wire technique, forceps technique, basket retrieval device and cook fragment retriever[1]–[3],[13],[14]. Balloon technique is the most common used method in previous reports[1],[2]. However, it is only suitable when the dislodged stent remains over the angioplasty wire. The two-wire technique is quite easy to perform, and yet it does not suit the situation when the lost stent has moved out of the guide-wire. Besides, it may not often be successful because it is hard to make the distal ends of the wire remain intermingled in order to exert enough force to extract the lost stent. By comparison, due to the larger sizes of the apparat, the forceps, basket retrieval device and cook fragment retriever would be used in the aorta, femoral, iliac and other similarly large arteries.

In conclusion, the gooseneck snare is an effective apparatus to retrieve the dislodged coronary stent when it migrates into the renal artery.

## References

[1] Brilakis ES, Best PJ, Elesber AA, Barsness GW, Lennon RJ, Holmes DR (2005). Incidence, retrieval methods, and outcomes of stent loss during percutaneous coronary intervention: a large single-center experience. Catheter Cardiovasc Interv.

[2] Eggebrecht H, Haude M, von Birgelen C, Oldenburg O, Baumgart D, Herrmann J (2000). Nonsurgical retrieval of embolized coronary stents. Catheter Cardiovasc Interv.

[3] Foster-Smith KW, Garratt KN, Higano ST, Holmes DR (1993). Retrieval techniques for managing flexible intracoronary stent misplacement. Cathet Cardiovasc Diagn.

[4] Alfonso F, Martinez D, Hernández R, Goicolea J, Segovia J, Fernández-Ortiz A (1996). Stent embolization during intracoronary stenting. Am J Cardiol.

[5] Elsner M, Peifer A, Kasper W (1996). Intracoronary loss of balloon mounted stents: successful retrieval with a 2 mm-“Microsnare”- device. Cathet Cardiovasc Diagn.

[6] Cantor WJ, Lazzam C, Cohen EA, Bowman KA, Dolman S, Mackie K (1998). Failed coronary stent deployment. Am Heart J.

[7] Kobayashi Y, Nonogi H, Miyazaki S, Daikoku S, Yamamoto Y, Takamiya M (1996). Successful retrieval of unexpanded Palmaz-Schatz stent from left main coronary artery. Cathet Cardiovasc Diagn.

[8] Veldhuijzen FL, Bonnier HJ, Michels HR, el Gamal MI, van Gelder BM (1993). Retrieval of undeployed stents from the right coronary artery: report of two cases. Cathet Cardiovasc Diagn.

[9] Juszkat R, Dziarmaga M, Zabicki B, Bychowiec B (2007). Successful coronary stent retrieval from the renal artery. Cardiol J.

[10] Beregi JP, Mauroy B, Willoteaux S, Mounier-Vehier C, Rémy-Jardin M, Francke J (1999). Anatomic variation in the origin of the main renal arteries: spiral CTA evaluation. Eur Radiol.

[11] Turba UC, Uflacker R, Bozlar U, Hagspiel KD (2009). Normal renal arterial anatomy assessed by multidetector CT angiography: Are there differences between men and women?. Clin Anat.

[12] Koseoglu K, Parildar M, Oran I, Memis A (2004). Retrieval of intravascular foreign bodies with goose neck snare. Eur J Radiol.

[13] Bogart DB, Jung SC (1999). Dislodged stent: a simple retrieval technique. Catheter Cardiovasc Interv.

[14] Douard H, Besse P, Broustet JP (1998). Successful retrieval of a lost coronary stent from the descending aorta using a loop basket intravascular retriever set. Cathet Cardiovasc Diagn.

